# Supplementary material for: Recognition of intrinsic values of sentient beings explains the sense of moral duty towards global nature conservation
Source: PLoS One. 2022 Oct 27;17(10):e0276614. doi: 10.1371/journal.pone.0276614 (PMC9612441; doi:10.1371/journal.pone.0276614)
Supplement: Attachment — Submitted filename: Review for PLOSONE.docx [file pone.0276614.s002.docx]

Review for PLOSONE

“For its own sake: intrinsic values explain the moral duty towards nature conservation better than relational values”

This article sets forth an empirical investigation into the theoretical ground for persons’ sense of duty toward the conversation of nature, and, related, identify the best theoretical predictors that persons hold a sense of duty to the conversation of nature.

As a general point, I’m pleased to see work in experimental philosophy targeting questions within environmental philosophy. Results from this type of work are worth generating and considering. Regrettably, though, I can’t recommend this paper for publication.

There are, as far as I can tell, no problems with the basic methodology and application of appropriate statistical tools. But the substance of the survey and the inferences drawn regarding the value of nature, I don’t think are successful.

The problem traces foundationally to the way in which the authors attempt to reveal respondents’ theoretic commitments to relational values. To be sure, the authors should be commended for detailing the Muracian framework, how they understand her accounting of relational values, as well as their acknowledging that questions of interpretation remain. Ultimately, though, I don’t think they’ve done enough in the set-up with their study to ensure that, IF respondents held something like a relational value view for conserving nature (or, perhaps, in keeping with the authors’ formulation, if relational values were psychological/emotional motivators for environmental protection), that their questions would demonstrate that.

Take first the authors’ version of the last person though experiment. As the original thought experiment intended, it’s perfectly reasonable to view those who judge it wrong for the last person to destroy the remaining living world to extend intrinsic value to that world. And we should expect to see consistency across this and the specific intrinsic value questions. But it’s not at all clear (in fact I’m skeptical) that either the last person thought experiment or the abstract questions regarding humans’ dependence on nature will prompt respondents to answer in a way reflective of their holding a relational value view of nature.

As I see it, the authors are asking too much of respondents by posing the questions as they do. For those persons who value in a way that is relational, the specific parts of nature and their unique connection to it is essential to making visible that valuing. A general question such as “I have a strong affinity towards nature and ecosystems which forms part of my sense of identity” strikes me as a question that many who may hold a relational value view of nature will not elicit a positive response. And the reason for this is that the respondent has to themselves recognize their own particular relational valuing from the abstract question.

Consider, for example, an avid and moderately enlightened duck hunter. This duck hunter has been hunting since childhood with friends and family. She has grown to rely on the duck for special occasion meals. She also has been a vocal proponent of wetlands protection to ensure the future of her being able to duck hunt. But when presented with the abstract question about having a strong affinity towards nature and ecosystems, the respondent has to work to make the connection between their relational valuing of wetlands and a positive response to that question. If, moreover, the duck hunter is contemptuous of most environmentalism (a very real possibility), their scoring of that question may be very low, despite them being a good example of a relational valuing of nature. The same, of course, would be true for the duck hunter’s treatment of the last person thought experiment. The implication, then, is that a failure to find respondents answering in ways consistent with a relational value framework may, plausibly, be a mirage; they do value nature on a relational basis, and thus seek nature conversation, but not GENERALLY as the question probes. They do it for their own particular parts of nature that they have a specific relationship with.

Notably, the authors gesture to concerns that relate to the above. First, they note that RV theorists highlight the context of valuing (p. 13). Second, they acknowledge that respondents may have difficulty understanding what relational values are (p. 15). For the first of these, the authors attempt controls for age and gender, where they find no statistical change. For the second, the authors suggest that probing for conservation along a temporal dimension would be useful. But neither of these points really address the essence of the problem outlined above.

In the controls for age and gender, it’s suggested that these would be proxies for the effects of context. But that doesn’t seem correct—see again the duck hunter case (and note that that is just a single illustration of the effect of one specific context). Regional and cultural factors are seemingly the more appropriate variables, but it’s important to keep in mind the issue centers on how to pose questions that optimize respondents’ ability to connect their particular human-natural world relationship with an inclination to protect the natural world (aside—as signaled above, I think there is also an interesting question that arises whether conservation of nature is derivatively local rather than global in a RV framework, again, countering the ability of the last person thought experiment to reveal respondents’ commitments to nature conservation within that framework). This basic point also plagues the temporal dimension idea for addressing respondents’ understanding of RV. The problem is not to ensure posing questions that attend to the temporal dimension of protecting nature; the worry is that the questions do not connect with the unique relationships respondents might have with parts of the natural world, showing them lacking relational values when in fact they have them and would seek (certain?) environmental protections were that connection made.

One small final point. The authors appear to use interchangeably the language of “explanatory” and “predictor.” Explanatory factors are (typically) temporally sensitive—the explanans come before the explanadum. For predictors, that need not be the case. In a circumstance like this, where certain statistical relationships are introduced to establish predictors, it’s probably not problematic to go further and assert they are explanatory. But that is an additional inference.
