# Supplementary material for: Recognition of intrinsic values of sentient beings explains the sense of moral duty towards global nature conservation
Source: PLoS One. 2022 Oct 27;17(10):e0276614. doi: 10.1371/journal.pone.0276614 (PMC9612441; doi:10.1371/journal.pone.0276614)
Supplement: Attachment — Submitted filename: Response to reviewers_final.docx [file pone.0276614.s003.docx]

Dear Professor Brigandt,

Please find herewith a revised version of our manuscript entitled 'Recognition of intrinsic values of sentient beings explains the sense of moral duty towards global nature conservation'. We thank the reviewers for their comments. They were very insightful and allowed us to improve the article. We have answered all the comments and acted upon the suggestions by the reviewers.

We hope that you will agree with us that the article has been substantially improved and that you will find it now suitable for publication.

Thank you.

Regards,

Lan Tianxiang

**Reviewer 1**

Comment 1: This article sets forth an empirical investigation into the theoretical ground for persons’ sense of duty toward the conversation of nature, and, related, identify the best theoretical predictors that persons hold a sense of duty to the conversation of nature.

As a general point, I’m pleased to see work in experimental philosophy targeting questions within environmental philosophy. Results from this type of work are worth generating and considering. Regrettably, though, I can’t recommend this paper for publication.

There are, as far as I can tell, no problems with the basic methodology and application of appropriate statistical tools. But the substance of the survey and the inferences drawn regarding the value of nature, I don’t think are successful.

The problem traces foundationally to the way in which the authors attempt to reveal respondents’ theoretic commitments to relational values. To be sure, the authors should be commended for detailing the Muracian framework, how they understand her accounting of relational values, as well as their acknowledging that questions of interpretation remain. Ultimately, though, I don’t think they’ve done enough in the set-up with their study to ensure that, IF respondents held something like a relational value view for conserving nature (or, perhaps, in keeping with the authors’ formulation, if relational values were psychological/emotional motivators for environmental protection), that their questions would demonstrate that.

Take first the authors' version of the last person thought experiment. As the original thought experiment intended, it's perfectly reasonable to view those who judge it wrong for the last person to destroy the remaining living world to extend intrinsic value to that world. And we should expect to see consistency across this and the specific intrinsic value questions. But it's not at all clear (in fact I'm skeptical) that either the last person thought experiment or the abstract questions regarding humans' dependence on nature will prompt respondents to answer in a way reflective of their holding a relational value view of nature.

As I see it, the authors are asking too much of respondents by posing the questions as they do. For those persons who value in a way that is relational, the specific parts of nature and their unique connection to it is essential to making visible that valuing. A general question such as "I have a strong affinity towards nature and ecosystems which forms part of my sense of identity" strikes me as a question that many who may hold a relational value view of nature will not elicit a positive response.

**Response**: Thank you for the insightful comments that helped us to improve the manuscript.

We agree with reviewer 1 that certain survey items may not elicit the intended response from participants under certain circumstances. We therefore accounted for the alternative explanation to our results by reviewer 1 by analysing the sensitivity of our results to item removal. We also toned down the discussion of the results by removing sentences throughout the paper that may be interpreted as critical to the RV theory.

Specifically, regarding this comment, we acknowledge the possibility that the item "I have a strong affinity towards ____ which forms part of my sense of identity" may not measure RV well. We thus tested the sensitivity of the results by removing the item "I have a strong affinity towards ____ which forms part of my sense of identity" and repeating the statistical analysis.

**Action in manuscript**

[Result of the sensitivity analysis, lines 290-293]

“We also assessed whether the statement “I have a strong affinity towards ___ which forms part of my sense of identity” measures RV well by repeating the analyses after removing the affinity & identity statement for all three moral patient types. The results did not change.”

[Lines 319-320]

“When repeating the analysis after removing the affinity & identity statements, the AIC values of models for scores of RV statements increased slightly. The AIC ranking of the models did not change.”

Comment 2: Consider, for example, an avid and moderately enlightened duck hunter. This duck hunter has been hunting since childhood with friends and family. She has grown to rely on the duck for special occasion meals. She also has been a vocal proponent of wetlands protection to ensure the future of her being able to duck hunt. But when presented with the abstract question about having a strong affinity towards nature and ecosystems, the respondent has to work to make the connection between their relational valuing of wetlands and a positive response to that question. If, moreover, the duck hunter is contemptuous of most environmentalism (a very real possibility), their scoring of that question may be very low, despite them being a good example of a relational valuing of nature. The same, of course, would be true for the duck hunter's treatment of the last person thought experiment. The implication, then, is that a failure to find respondents answering in ways consistent with a relational value framework may, plausibly, be a mirage; they do value nature on a relational basis, and thus seek nature conversation, but not GENERALLY as the question probes. They do it for their own particular parts of nature that they have a specific relationship with.

**Response**: Thank you for the suggestion. We acknowledge that our survey questions elicit more of the relational values arising from global, fundamental dependence than that of local (and arguably functional) dependence. In the revised script, we thus added this qualification and pointed out that RV likely still play an important role in the care for local environment.

**Action in manuscript**

[lines 377-389, emphasis added in this letter] We added a paragraph explaining the limitation of not focusing on local concerns and emphasised that the strength or weakness of a kind of environmental value in explaining the sense of moral duty is always context-dependent.

“We acknowledge, however, that limitations come along with the above advantages for the scope chosen. One important trait of RV is that they may be rooted in people’s cultural customs, daily practices and unique experiences of interacting with the local environment – in short, the situatedness of knowledge. The survey items and the moral judgment scenario are geared towards measuring global concern for the future, hence they may not measure well meanings attached to nature via unique experiences or concerns for the local environment. For instance, for some indigenous communities, the perception of nature is intertwined with cultural values and experiences linked to the local environment. Hence, they may not resonate with a general, abstract global conservation agenda not connected to their local knowledge and unique experiences. This does not make those people any less caring about the environment. The upshot of the discussion is that just like the sense of moral duty, the practical significance of recognising RV is also context-dependent. There may not be a one-size-fits-all type of moral values that explains the sense of moral duty towards nature conservation regardless of context.”

[lines 398-403] ”Third, this study only concerns whether RV explain people’s sense of duty for future nature, rather than the present nature. We thus do not deny the prospect that RV may explain well the sense of duty to prevent sudden, drastic changes to the present nature (especially locally) such as deforestation or destructive fishing practices, which destroy the ecosystem with which present humans have a morally significant relationship.”

Comment 3: In the controls for age and gender, it’s suggested that these would be proxies for the effects of context. But that doesn’t seem correct—see again the duck hunter case (and note that that is just a single illustration of the effect of one specific context). Regional and cultural factors are seemingly the more appropriate variables, but it’s important to keep in mind the issue centers on how to pose questions that optimize respondents’ ability to connect their particular human-natural world relationship with an inclination to protect the natural world (aside—as signaled above, I think there is also an interesting question that arises whether conservation of nature is derivatively local rather than global in a RV framework, again, countering the ability of the last person thought experiment to reveal respondents’ commitments to nature conservation within that framework). This basic point also plagues the temporal dimension idea for addressing respondents’ understanding of RV. The problem is not to ensure posing questions that attend to the temporal dimension of protecting nature; the worry is that the questions do not connect with the unique relationships respondents might have with parts of the natural world, showing them lacking relational values when in fact they have them and would seek (certain?) environmental protections were that connection made.

**Response**: Thank you for the suggestion. We acknowledge this drawback, and thus clarified section that this study only measures the sense of duty for future *global* conservation efforts not specific to the unique experiences of the individuals. We also explained why this particular focus is important.

**Action in manuscript**

We added a paragraph to explain the practical significance of choosing to focus on the sense of moral duty towards a general global environment in the future in this study.

[Lines 360-376, emphasis added in this letter]

In this study, we chose to focus on the sense of moral duty towards the global natural environment, at a time scale of 50-100 years into the future, rather than the present, local environment. We believe that the scope chosen has high practical relevance. Today, neoliberal market favours the transfer of adverse environmental impacts from the developed to the developing region (40) through export, outsourcing and so on (41-43). Climate change also imposes greater risks of temperature and rainfall extremes on the tropical region (44), while developed economies in Europe, North Asia and North America emit greenhouse gases in quantity disproportionate to their population. This means for many people with a high environmental footprint, their impacts are much more manifest outside their home region. Thus, a sense of duty towards global conservation is important so that those people may have a genuine concern for the damages happening far away from them. Second, psychological connectedness with a party may decrease with temporal distance (45), while the effects of environmental conservation usually have substantial time lags. For instance, even after greenhouse gas emission ceases, global temperature and sea level are set to rise for centuries if not millennium (46, 47); a similar time lag in response to climate change is predicted for terrestrial ecosystems as well (48). This effectively means a lot of conservation actions are more for the future Earth, and it is thus important for one to have a sense of moral duty directed at future entities despite the effect of temporal distance.

Comment 4: One small final point. The authors appear to use interchangeably the language of "explanatory" and "predictor." Explanatory factors are (typically) temporally sensitive—the explanans come before the explanadum. For predictors, that need not be the case. In a circumstance like this, where certain statistical relationships are introduced to establish predictors, it's probably not problematic to go further and assert they are explanatory. But that is an additional inference.

**Response**: We have edited the script accordingly and we thank the reviewer for sharing the knowledge.

**Reviewer 2**

Comment 1: My overall impression of this paper is that it is (as far as I can tell) a competently conducted study of the importance of relational values in predicting people's moral attitudes towards nature and provides some evidence that appears to be in conflict with some earlier studies. It is mostly well written with a clear and easy to follow structure. A lot of the questions I found myself thinking about when reading this paper where subsequently brought up and dealt with in the discussion section. I think this paper is in a condition that is publishable as it is and the comments and remarks I have are minor.

I think the main charge against this approach to relational values turns on how the concept is operationalised and tested at a very general level. The commitment to a weaker conception of relational values is convenient clearly and possibly is what allows for a study like this to be conducted but it seems to be based on assumptions that are antithetical to what many would believe to be significant about the turn to relational values. Especially the challenge to a sharp dichotomy between humans and nature is important as it would make a lot of the questions posed in this study appear somewhat meaningless. In the relational values framework, as I understand it, it just doesn't make sense to talk of things like 'dependence' for example and the very idea of conservation becomes highly suspicious (and in practice often a vehicle for oppression). Now the authors flag an awareness of this but dismiss stronger varieties of the concept. Now, I am willing to accept the setup here. Testing stronger conceptions of relational values is perhaps more difficult to do rigorously.

**Response**: Thank you for the positive feedback and constructive comments. We further emphasised in the discussion that the chosen scope and focus of this study comes with limitations, so that the results are not interpreted as an unqualified challenge to the usefulness of the relational value concept.

**Action in manuscript**

[lines 377-389] We added a paragraph explaining the limitation of not focusing on the general global concerns for the environment which may not be connected to the situated knowledge of the people.

“We acknowledge, however, that limitations come along with the above advantages for the scope chosen. One important trait of RV is that they may be rooted in people’s cultural customs, daily practices and unique experiences of interacting with the local environment – in short, the situatedness of knowledge. The survey items and the moral judgment scenario are geared towards measuring global concern for the future, hence they may not measure well meanings attached to nature via unique experiences or concerns for the local environment. For instance, for some indigenous communities, the perception of nature is intertwined with cultural values and experiences linked to the local environment. Hence, they may not resonate with a general, abstract global conservation agenda not connected to their local knowledge and unique experiences. This does not make those people any less caring about the environment. The upshot of the discussion is that just like the sense of moral duty, the practical significance of recognising RV is also context-dependent. There may not be a one-size-fits-all type of moral values that explains the sense of moral duty towards nature conservation regardless of context.”

Comment 2: I mostly wondering whether these prompts actually engage the right kind of thing in test. For example, how are respondents understanding 'dependence'? Is it possible to think that dependence is understood narrowly by respondents and that there might be other 'non-dependence' relationships that are the objects of the relational values and that the experimental setup is blind to these?

**Response**: Thank you for the suggestion. We acknowledge that the notion of 'dependence' is not clearly defined in the survey. We have thus pointed it out in the discussion section.

**Action in manuscript**

[Lines 420-423] "One more point of note is that “dependence” in the survey of this study was not explicitly defined for conciseness. We recognise the possibility that respondents may have different interpretations of the term from each other. Future studies may provide detailed explanations and descriptions of the key concepts used in the survey."

Comment 3: Regarding the concept of (personal) identity that is supposed to elicit from respondents relational values I am wondering how respondents understand these constructs. This is a way of operationalising the concept, of course, but it seems potentially narrow. I'm not entirely convinced from the discussion on relational values in the paper that this is an iron clad construction for getting at these values.

**Response**: Thank you for the suggestion. We acknowledge the possibility that the item "I have a strong affinity towards ____ which forms part of my sense of identity" may not measure RV well. We thus tested the sensitivity of the results by removing the item "I have a strong affinity towards ____ which forms part of my sense of identity" and repeating the statistical analysis.

We also recognise that this study presented only one of the many ways of operationalising RV, and pointed this out in the discussion section.

**Action in manuscript**

[Result of the sensitivity analysis, lines 290-293]

“We also assessed whether the statement “I have a strong affinity towards ___ which forms part of my sense of identity” measures RV well by repeating the analyses after removing the affinity & identity statement for all three moral patient types. The results did not change.”

[Recognising the limitation in operationalisation, lines 392-395]

"The notion that dependence is an important element of RV is derived from our interpretation of the literature. The same applies to the survey items used in this study. We recognise that diverse interpretations of the concept may exist, and they may show different results when operationalised and measured."

Comment 4: Sometimes the authors seem to conflate 'conservation efforts' with having certain moral attitudes towards nature. It is a minor thing, maybe, but I am not exactly sure these are the same necessarily. Conservation could perhaps be understood as something more narrow than simply caring about nature. I.e., one might think that conventional conservation does not adequately protect nature, or are even counter productive.

Comment 5: Page 4: The notion of 'exploitative' anthropocentrism is not clear to me. Why is it exploitative? In virtue of being anthropocentric? Or is it a kind of anthropocentrism that is exploitative? I suspect the former, but nonetheless. I'm not sure these qualifiers add to the clarity of the paper. Something similar could be said about 'altruistic non-anthropocentrism.

**Response**: Regarding the above two comments, we have edited the script accordingly and we thank the reviewer for pointing out the need for clarification.

**Action in manuscript**

[Lines 70] The second half of the sentence "…which deviates from the assumed dualism between exploitative anthropocentrism and altruistic non-anthropocentrism in the traditional sense" is removed.

[Lines 144-146] "The word 'conservation' refers to a generic sense of wanting to benefit, protect, or reduce and mitigate one's impacts on nature, to avoid the debate of what constitutes legitimate or productive conservation efforts." This sentence is added.

Comment 6: Page 3: Concerning the discussion on intrinsic versus instrumental values. I guess subjectivists about values will not think of intrinsic values as being ‘inherently possessed’ (maybe, I’m not sure what ‘inherently possessed’ is supposed to entail). For the subjectivist even intrinsic values typically requires a subject of some sort valuing the things (but doing so in a certain way).

**Response**: Thank you for the suggestion. We clarified the definition of intrinsic values used in this study and pointed out the metaethical commitment of the definition used.

**Action in manuscript**

[Lines 170-175] "The term ‘intrinsic values’ also needs clarification... In some other occasions, it may refer to non-extrinsic values. This means values that something has in virtue of its intrinsic properties, ‘independently of any connection to any valuable thing’.”

[Lines 179-188, emphasis added in this letter] "This study interprets intrinsic values as non-extrinsic values, for two reasons. Theoretically, defining intrinsic values as values that exist independent of an entity’s external relations (which has a moral realist overtone) contrasts well with RV which is dependent of an entity’s relation with humans. We may thus be reasonably confident that the intrinsic values survey items developed based on this definition are measuring a construct sufficiently distinct from RV. Practically, it is also a broader concept compared to other definitions, and it overlaps very much with the notion of non-instrumental values which the term “intrinsic values” most commonly refers to – recall that RV are proposed for overcoming the “dichotomy” between intrinsic values and instrumental values, for instance. This means survey items developed based on this definition may better resonate with how the term is popularly used.”

Comment 7: Page 7: It seems to me that trying to investigate whether RV are “able” to drive a sense of moral duty towards nature is perhaps too strong. The framework might be the (normatively) right one, but still not be intuitive for many contemporaries. If they could be educated somehow this might change. Minimally it seems one could approach the relational values framework as a framework that has normative force (although not necessarily being the intuitive one for people in general) or as (also) having some descriptive content. I.e., it better reflects how people think.

**Response**: Thank you for pointing this out. We changed “drive” to “explain” to avoid overly strong assumptions about RV’s role in moral intuition formation.

**Action in manuscript**

[Lines 130] " Does recognising the RV of nature explain people’s sense of moral duty towards global nature conservation for the future?”

[Lines 136] "H1: Recognising the RV of nature explains people’s sense of moral duty towards global nature conservation for the future.”

Comment 8: P16 (line 319 and onwards). I’m not sure what the authors are trying to say here. They at least come very close to transgressing the line between the normative and descriptive. The results show that intrinsic values are enough to provide people (in the survey) with reasons for protecting nature. Or at least a sense of moral responsibility towards nature. It does not seem to adjudicate between the philosophical positions as philosophical positions. Although it might, as the authors seem to say, provide some idea of what kind of arguments might be persuasive vis a vis the general public.

**Response**: Thank you for pointing this out, the issue at line 319 is a mistake on our end. The paragraph has been modified to make clear that the results bear direct implication only to the descriptive, not the normative.

**Action in manuscript**

[Lines 336-345, emphasis added in this letter] "At a practical level, the notion that recognising the intrinsic values of sentient beings is a major predictor of people’s sense of moral duty towards global nature conservation for the future seems to weigh in favour of traditional sentientism. The fact that intrinsic values best predict the moral intuition to protect future nature means it is less likely that traditional sentientism distracts people from caring for nature (37). Though empirical findings as such cannot offer theoretical protection for sentientism, they may provide reason to be confident that belief in sentientism may work as well as, or even better than, holistic theories in explaining and eliciting laypeople’s sense of moral duty to conserve nature. At the very least, the results provide a reason to doubt the intuitiveness amongst the public of arguments like ‘animal-welfare ethics have been either irrelevant to conservation and environmental concerns or an impediment to them’, or ‘conservationists qua conservationists are simply not concerned about the welfare of individual organisms’ (10).”

Comment 9: To conclude, I really liked this paper and I think it is a nice and well-written contribution to the discussion on relational values and well deserves to be published. With some minor adjustments and clarifications the paper could be slightly improved.

**Response**: Thank you again for the positive feedback and constructive comments.
